# Resection and reconstruction of huge tumors in the chest wall

**DOI:** 10.1186/s13019-022-01877-9

**Published:** 2022-05-12

**Authors:** Zhibing Dai, Maierdanjiang Maihemuti, Yachao Sun, Renbing Jiang

**Affiliations:** Department of Bone and Soft Tissue, Affiliated Tumor Hospital of Xinjiang Medical University, Urumqi, Xinjiang China

**Keywords:** Chest wall tumor, Resection, Reconstruction, Multidisciplinary, Perioperative period

## Abstract

**Objective:**

To evaluate the experience and effects of resection and reconstruction of 4 cases of huge tumors in the chest wall.

**Methods:**

The clinical data of 4 patients with huge tumors in the chest wall from July 2015 to January 2020 were collected and analyzed. There were 2 males and 2 females.Chondrosarcoma was diagnosed in 2 cases, giant cell tumor was diagnosed in 1 case,and metastasis from breast cancer was diagnosed in 1 case.All patients underwent extensive tumor resection and had thoracic exposure after tumor resection.Two patients underwent reconstruction with mesh and titanium mesh, and the incision was closed directly.The third patient underwent reconstruction with mesh and latissimus dorsi flap,and the fourth patient underwent reconstruction with mesh,titanium mesh and latissimus dorsi flap.

**Result:**

One patient had incision infection after operation,which resolved after debridement.All patients were followed up for 2–6 years, no tumor recurrence or metastasis was noted during follow-up.None of patients had abnormal breathing, dyspnea or other physical discomfort.

**Conclusion:**

It is difficult to resect the huge tumors in the chest wall,and it is more reasonable and safer to choose a reconstruction method using mesh and titanium mesh.The latissimus dorsi flap can achieve good results in repairing soft tissue defects.Close perioperative management and multidisciplinary team discussions can help to achieve better curative effects.

## Background

Huge tumors occurring in the chest wall include primary tumors, malignant tumors that invade the chest wall directly or metastatic lesions of the chest wall [1, 2].Because there are few soft tissues in the chest wall and the barrier effect is weak, tumors can grow very large and can invade the skin.These huge tumors can also invade important anatomical structures in the thoracic cavity, making them difficult to treat. After tumor resection, huge bone and soft tissue defects can occur, exposing the thoracic organs.It is therefore necessary to reconstruct and restore the chest wall to protect the lung tissue and internal organs, prevent chest wall hernia and restore normal breathing.Because the chest wall has less soft tissue, After tumor resection, the skin and muscle defects are often large defect. The application of latissimus dorsi flap to cover the wound can promote early incision healing, allowing patients to receive adjuvant radiotherapy, chemotherapy or other treatment more quickly.Herein,we describe our clinical experience regarding the resection of huge tumors in the chest wall and the effects of subsequent reconstruction.

## Clinical data

This study included 4 patients with giant tumors in the chest wall who were treated from July 2015 to January 2020.There were 2 males and 2 females, with an average age of 48.5 years.The size of the tumor was approximately 10 × 10 cm -20 × 15 cm.The tumor invaded the ipsilateral 2 ribs and partial sternum in 1 case, the ipsilateral 3 ribs and partial sternum in 1 case, the bilateral 2 ribs and sternal manubrium in 1 case, and the ipsilateral 4 ribs and middle and upper sternumin in 1 case.Chondrosarcoma in 2 cases, giant cell tumor in 1 cases, and metastasis in 1 cases. Huge lump in the chest wall was observed. All patient reported that the lumps were painful.The pain was severe when the mass was pressed and some patients had intercostal radiation pain (Table 1).

**Table 1:** Clinical data of the patients and tumors

Age(years)	25	63	52	54
Sex	Male	Female	Male	Female
Tumor size(cm)	10 × 10	17 × 13	20 × 15	18 × 15
Invasive extent	Left 1st,2nd ribs, partial sternum	Right 4th-6th ribs, partial sternum	Bilateral 1st,2nd ribs, manubrium sterni	Lefe 1st-4th ribs, middle and upper sternum
Pathology	Giant cell tumor of bone	Chondrosarcoma	Chondrosarcoma	Breast cancer metastasis
Reconstruction	Mesh, Titanium mesh	Mesh, Titanium mesh	Mesh, Latissimus dorsi myocutaneous flap	Mesh, Latissimus dorsi myocutaneous flap
Operation time	5 h	7 h	10 h	6 h
Blood loss	800 ml	1600 ml	4800 ml	2300 ml
Observation time	5 years	6 years	2 years	2 years

## Method

### Preoperative preparation

Patients received *X*-ray, enhanced CT, and enhanced MR, and PET-CT examinations to assess whether the patients had other site metastases. The pathology was confirmed by needle biopsy. The 3D printed models were designed and constructed according to imaging date. This aided in understanding the spatial location of the tumor and the adjacent anatomical structure.

Multidisciplinary discussion was conducted routinely before the operation. The Imaging department, anesthesiology department, thoracic surgery department, radiotherapy department, pathology department and ICU participated in the discussion. The position, and depth of the tumor, and the invaded tissues were determined according to CT and MR imaging. Clinicians delineated the boundary for tumor resection and determined which soft tissues, bone structures and lung tissues needed to be removed. A thorough analysis of the relationship between important blood vessels and tumors is required, especially with regard to the subclavian arteries and veins. If there was any fat observed between the tumor and blood vessels on imaging, the blood vessels could generally be completely preserved. If the blood vessel was obviously compressed and the diameter was narrow, freeing the blood vessel during the operation may not be feasible because the blood vessel might be rupture, causing massive bleeding. If the tumor invades the blood vessels, it is necessary to remove partial blood vessel wall or even replace the blood vessels. The barrier effect of pleural tissue is typically weak, and huge chest wall tumors often protrude into the chest. In addition, huge tumors often invade the lung tissue, necessitating resection Invasive lung tissue by thoracic surgeons. The anesthesiology department should prepare fully for the whole surgical process. After the operation, these patients should be sent to the ICU for close monitoring so that they can achieve the best possible recovery after the operation.

The chest wall participates in the respiratory system and protects thoracic organs. The integrity of chest wall soft tissue and bone scaffolds are essential for maintaining normal respiratory function.Because the soft tissue and bone defects were quite substantial after tumor resection. Most surgeons agreed that the incidence of pulmonary hernia could be high and that abnormal breathing would occur when defects with a diameter greater than 5 cm or four or more ribs were removed. In this situation, reconstruction of the chest wall is recommended [3–6].The defect of the chest wall was reconstructed with mesh, and then the titanium mesh was placed on the surface of the mesh for hard reconstruction. This combined reconstruction technique could not only protect the thoracic organs, but also prevent the occurrence of abnormal breathing. If the surrounding residual skin and muscle tissue could not cover the wound, it was covered with latissimus dorsi myocutaneous flap.

### Operation process

**Fig. 1:**
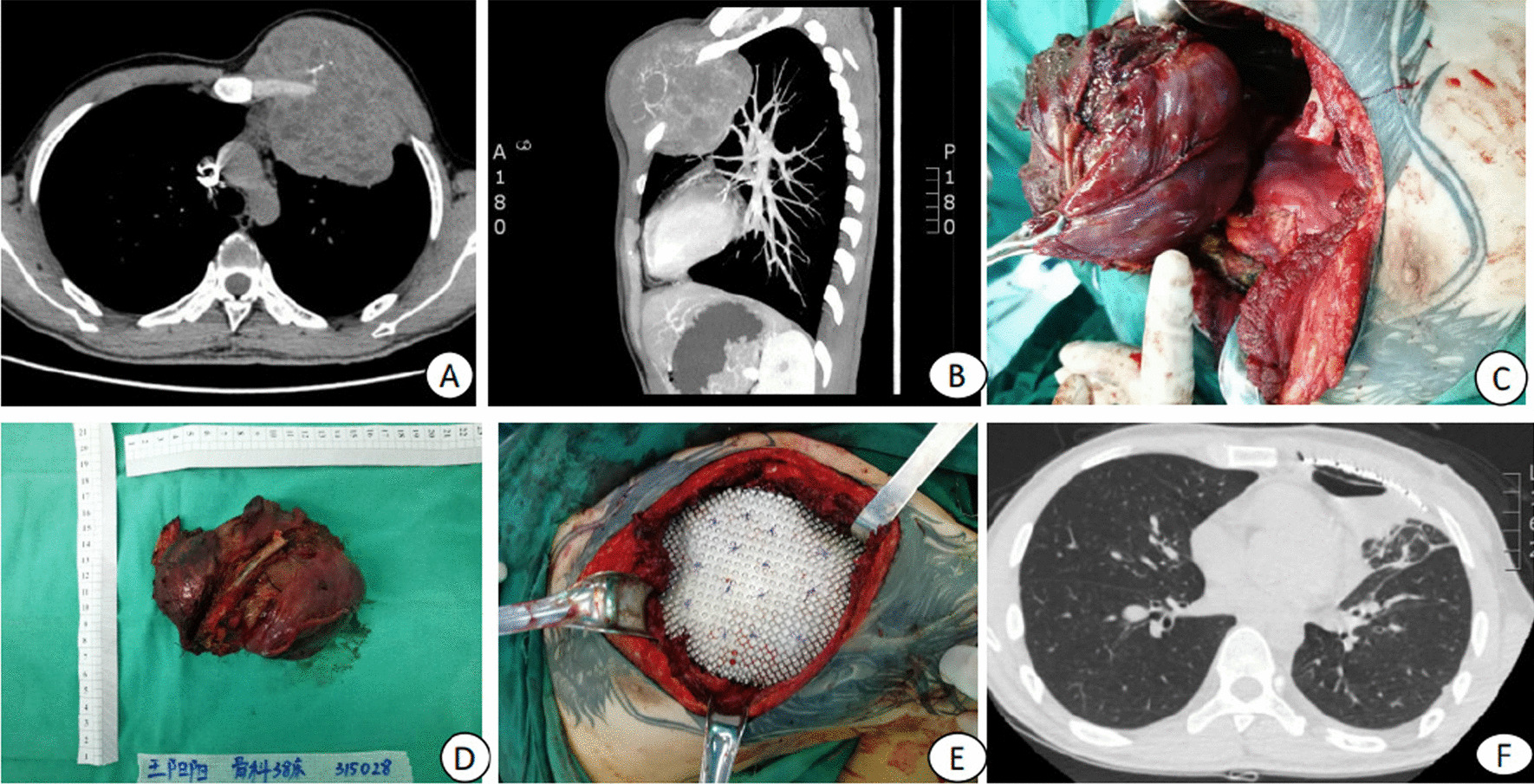
**A**-**B** CT showing a huge tumor protruding into the chest wall. **C** The invading lung tissue was resected during the operation. **D** Postoperative specimen. **E** Reconstruction chest wall with mesh and titanium mesh. **F** Postoperative CT showed that the lung recovered well

**Fig. 2:**
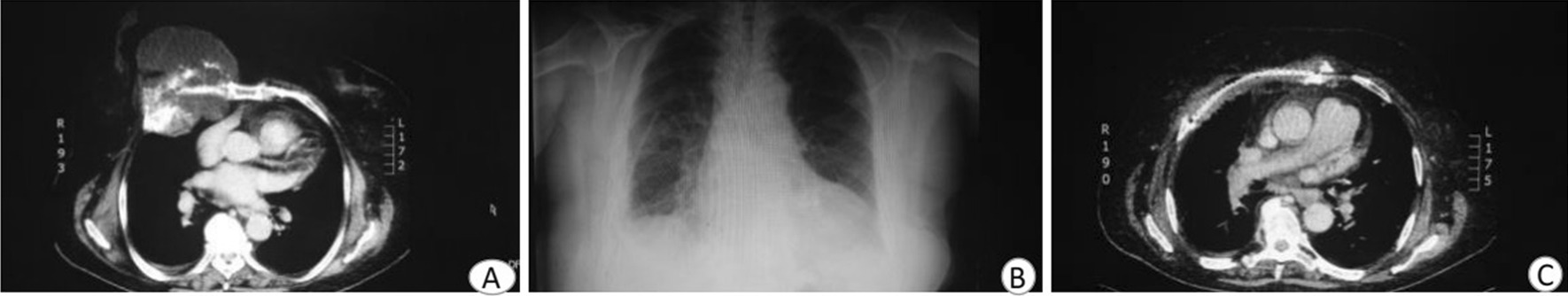
**A** The huge tumor in the right chest wall was observed that invaded the ribs and partially invaded the sternum. **B** Postoperative chest film,reconstruction by mesh and titanium mesh. **C** The position of the titanium mesh after the operation appeared to be good on CT, and the lung recovered well

**Fig. 3:**
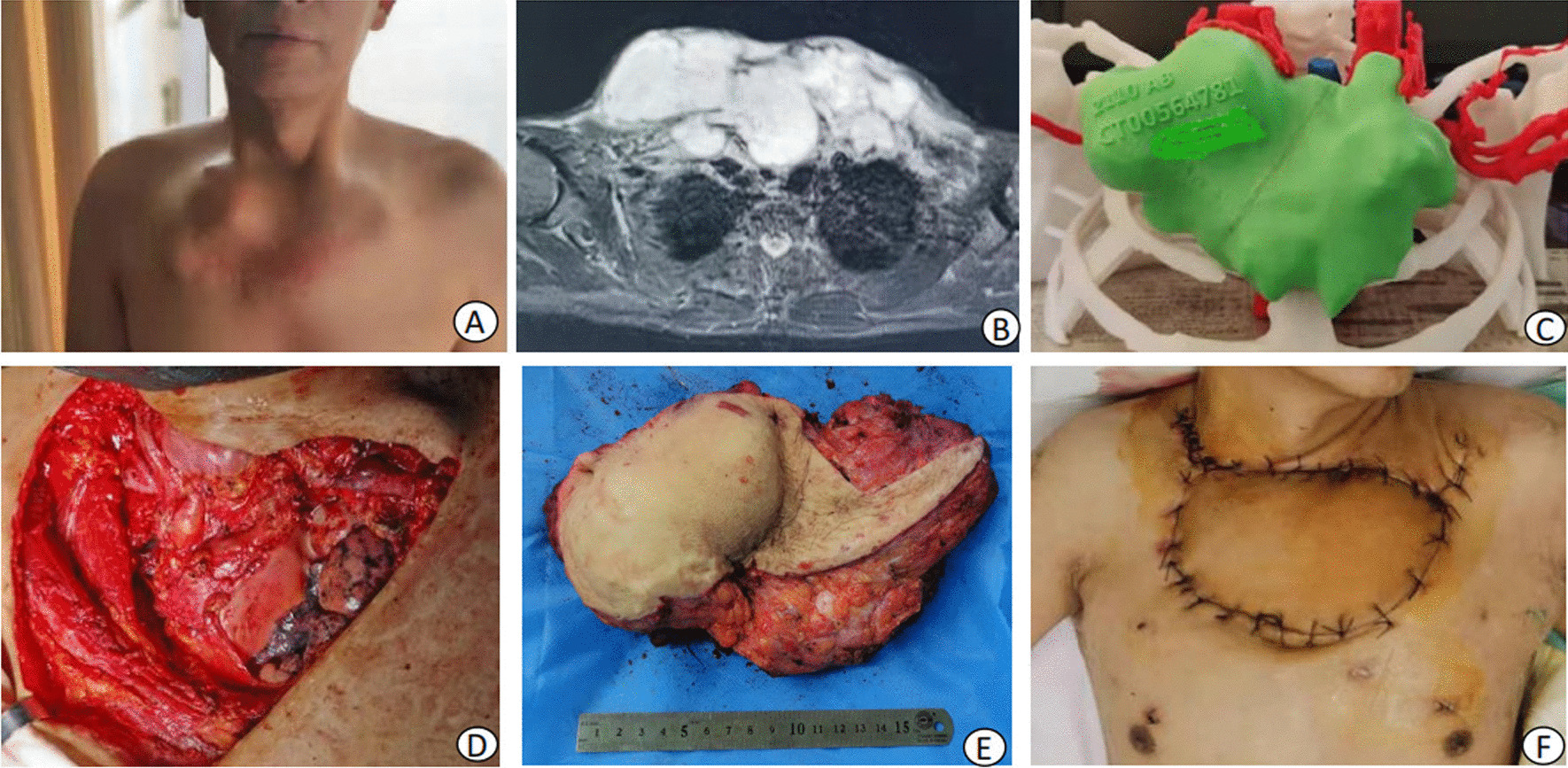
**A** Giant tumor in the upper chest wall. **B** MR showed that the tumor bilaterally invaded the clavicle, 1st and 2nd ribs, and the pleura, along with the anterior mediastinum and left subclavian vein. **C** 3D printing model showed a huge tumor in the anterior chest wall. **D** After tumor resection, the thorax was exposed bilaterally. **E** Tumor specimen. **F** The latissimus dorsi flap survived well at 3 weeks after the operation

**Fig. 4:**
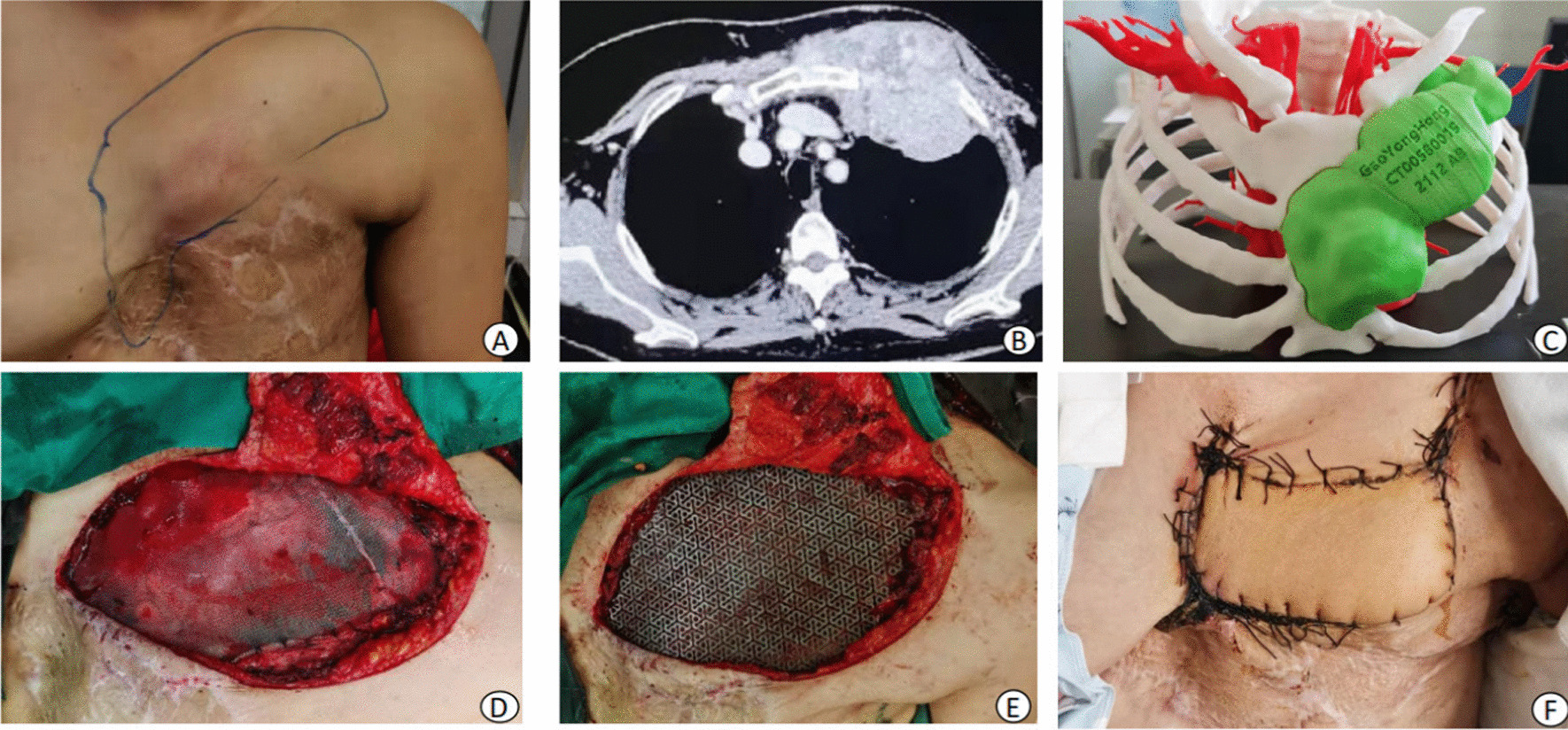
**A** A huge tumor on the left chest wall and an old scar on the left chest wall **B** Huge tumor on the left chest wall that invaded ribs 1–4, the sternum and lung tissue. **C** 3D printed model showing the huge tumor in the left anterior chest wall. **D** The chest wall defect was repaired with mesh **E** The titanium mesh on the polypropylene mesh. **F** The soft tissue defect was repaired with a latissimus dorsi myocutaneous flap, which grew well at one month postoperatively

Under general anesthesia, a spindle incision was made according to the long diameter of the tumor, and the skin tissue invading the tumor was removed at the same time.Bone and soft tissue were resected 3 cm away from the tumor, with the negative margin being the most critical. Because huge tumors often invade the thorax, ribs, pleura, and even some lung tissue, all of these components often need to be resected together to achieve R0 incisal margins. One patient had a giant cell tumor involving bone in the chest wall, and the tumor grew both inside and outside of the thorax. Two ribs, part of the sternum and some lung tissue were removed, and the chest wall defect was reconstructed with mesh and titanium mesh. Because there were few soft tissue defects, the stump of the pectoralis major muscle could be sutured to cover the titanium mesh, and the skin was sutured directly (Fig. 1). One patient with a giant chondrosarcoma on the right chest wall underwent resection of ribs 4, 5 and 6 and partial sternal resection. The pleura and part of the pericardium were also removed. The pericardium was sutured directly; the chest wall was reconstructed with mesh and titanium mesh. After tumor resection, it was found that the local subcutaneous tissue was thick, and the skin elasticity was good, so the wound was closed directly (Fig. 2).One patient had a giant chondrosarcoma on the upper chest wall that had recurred after surgery in another hospital. The maximum diameter of the tumor was 20 cm, involving the bilateral clavicles, bilateral first and second ribs and sternal manubrium. The tumor infringed on the subclavian vein, anterior mediastinum and bilateral pleura. The sternal manubrium, bilateral partial clavicles, bilateral 1st and 2nd ribs, and bilateral partial pleura were all resected. When part of the subclavian vein wall was removed, more bleeding occurred. After the vein wall was repaired, the bleeding could be controlled. During the operation the patient experienced a brief cardiac arrest, vagus nerve stimulation and cardiac compressions were required to maintain the circulation for approximately 10 s.The defect in the chest wall was repaired with mesh. Because there were many soft tissue defects, the incision could not be closed directly. Therefore, the wound was repaired by a latissimus dorsi myocutaneous flap (Fig. 3).One case of chest wall metastasis after breast cancer operation caused a huge tumor in the left chest wall. The tumor invaded the lung tissue and subclavian vein. During the operation, a length of the subclavian vein was resected, 2/3 of the left clavicle, the left 1st-4th ribs and the middle and upper segments of the sternum, and part of the lung lobe was removed. The defect in the chest wall was reconstructed with mesh and titanium mesh. The titanium mesh overlapped with the cutting edge of the rib or sternum by 1–2 cm. The titanium mesh was fixed to the surrounding structure. Because the wound could not be closed directly, a latissimus dorsi flap was used to repair the soft tissue defect (Fig. 4).

## Results

All patients were transferred to the ICU after the operation. Two patients were transferred back to the general ward on the second day, and one patient was transferred back to the general ward three days later. One patient with a giant chondrosarcoma on the upper chest wall underwent partial resection of the bilateral pleura and exposure of the bilateral thorax. After the operation, the patient experienced respiratory and circulatory instability, followed by atelectasis, pleural effusion and pulmonary infection. After treatment in the ICU for three weeks, the patient's condition stabilized, and he was transferred back to the ward. One patient with a chondrosarcoma on the right chest wall suffered from infection one week after the operation, which resolved after debridement. All patients were followed up for 2–6 years. At present, none of the patients have experienced tumor recurrence or metastasis, or abnormal breathing, dyspnea or other discomfort.

## Discussion

Malignant tumors include primary and secondary tumors of the chest wall. Primary sarcoma and recurrent breast cancer mostly invade the chest wall. Primary chest wall tumors are most common in patients with chondrosarcoma and fibrosarcoma, accounting for approximately 77.8% of all cases [7].The treatment principle of chest wall malignant tumors is to achieve a negative margin by radical resection, prolong the survival time, and reduce the mortality and postoperative recurrence rate [8].It is important to note that the edge of the tumor should not be sacrificed to narrow the resection range of the chest wall [9].For primary malignant tumors that are insensitive to radiotherapy and chemotherapy, clinicians should pay more attention to the surgical margin. The integrity of the chest wall structure is paramount for ensuring normal respiratory function. However, the chest wall structure is relatively weak, the barrier effect is not significant. Larger tumors often invade the skin, multiple ribs and the pleura even lung tissue. Because the subclavian artery and vein have different spatial locations, tumors in the upper chest wall invade the subclavian vein first. Because the vein wall was relatively weak, the risk of vascular injury is high during separation. If blood vessels was injured, there would be a lot of blood loss. If the subclavian vein is invaded by a tumor, a range of blood vessels must be resected. One patient with chondrosarcoma in the upper chest wall, the tumor had invaded the left subclavian vein. Thus, the surgeon resected part of the venous wall and subsequently repaired it. The blood loss volume was 4800 ml. Another patient with chest wall metastasis from breast cancer, the tumor had invaded the subclavian vein and could not be separated, so a range of blood vessels were removed. This patient experienced slight edema in the upper limb after the operation, but it did not affect function, and she had no obvious discomfort. Removing multiple ribs and lung tissue causes respiratory and circulatory instability during the operation. Therefore, we carried out a multidisciplinary discussion before the operation, and the anesthesiology department dealt well with any intraoperative emergencies that arose. Extensive chest wall resection has often led to serious complications and mortality in the past [10, 11]. With advancements in surgical technology, anesthesia, nursing care and rehabilitation, the perioperative mortality rate has been reduced gradually [12, 13].Therefore, multidisciplinary participation is essential. To achieve good function, it is necessary to rebuild bone stability, protect the thoracic organs, achieve normal respiratory function, prevent pulmonary hernia from affecting the patient’s respiratory and circulatory function, and provide an acceptable appearance [14]. A balance between the stability of anatomical structure and the maintenance of function should be consider to ensure the best results.

The best method for chest wall reconstruction depends on the size, location and depth of the defect [14]. For small defects that do not influence the stability of the chest wall, closure through the mesh and local soft tissue can be performed at one time.Large defects might cause abnormal breathing, which usually requires more solid reconstruction. Deschamps et al.[15] proposed that rigid reconstruction is not necessary for the defect of chest wall and lateral chest wall less than 5 cm in diameter, or the defect of dorsal scapula. The defect was located in the sternum and parasternal region, especially in the precordial area,hard reconstruction is very important to maintain cardiac function, to prevent chest wall collapse and to protect intrathoracic organs [16, 17].

Various repair materials are available that have their own advantages and disadvantages [18]. Physicians needed to weigh the benefits of each material and technology to prevent infection and other complications as much as possible. Mesh and soft tissue flaps are common materials for the reconstruction of chest wall defects. Other materials such as titanium related products, autologous bone and allogeneic bone can be used to reconstruct the chest wall.The ideal chest wall repair material should have the following characteristics: sufficient hardness to prevent abnormal movement of the chest; good biocompatibility to promote tissue growth; good flexibility to make a suitable shape; and no interference with follow-up imaging examinations as much as possible.[8]. Polypropylene mesh (Marlex) is commonly used because it is relatively inexpensive and has good affinity for tissue growth, but it lacks sufficient hardness to restrain abnormal breathing in extensive defects.The use of titanium mesh in chest wall reconstruction has several obvious advantages, such as its light weight, corrosion resistance, inertia, good flexibility, superior strength to weight ratio, good biocompatibility and magnetic compatibility. It has enough hardness to protect the chest wall and inhibit abnormal breathing.It could be applied to huge chest wall defects with good performance and safety [16, 19].

After the resection of huge tumors, the chest wall defects are quite large. We repaired chest wall defects with mesh first and then with titanium mesh. As time went on, scars formed around the mesh, which reestablished the negative pressure environment within the thoracic cavity, protecting the lung tissue and preventing adhesion of the lung tissue.The titanium mesh can achieve hard reconstruction and protect the thoracic organs. Composite reconstruction techniques of titanium mesh and mesh achieved a better effect. We used mesh and titanium mesh when reconstructing defects involving 2 or more ribs. In one patient with giant chondrosarcoma of the upper chest wall, the sternal manubrium, bilateral partial clavicles and bilateral first and second ribs were resected, and the chest wall defect was indeed quite large. This patient underwent a second operation due to tumor recurrence. The tissue adhesion was so severe in this case that it was difficult to separate. The operation took up to 10 h to complete, there was a large volume of blood lost, and respiratory and circulatory instability occurred during the operation. To reduce the operation time, hard reconstruction by steel plate or titanium mesh was not carried out. Mesh was used to repair the defect, and then a latissimus dorsi myocutaneous flap was applied to cover the wound. The patient was sent to the ICU for further treatment after the operation. In the early stage after the operation, we also worried about whether the patient would have abnormal breathing. As the patient recovered gradually, no respiratory dysfunction was noted. However, the lack of hard protection of the chest wall was a deficiency in this case. If intraoperative conditions permit, the reconstruction of bone defects with steel plates or titanium mesh might achieve better results.The coverage of chest wall soft tissue could be achieved by direct closure, skin transplantation, local advancement flap, pedicled myocutaneous flap or free flap.The skin, subcutaneous tissue and muscle tissue have large defects after the resection of the huge tumor in the chest wall, and there are mesh and titanium mesh plants. The use of myocutaneous flap can cover the wound and prevent infection at the same time.Latissimus dorsi myocutaneous flap was selected in 2 patients to repair soft tissue defects. There was no incision infection and the flap survived completely. Latissimus dorsi myocutaneous flap has obvious advantages in repairing huge chest wall soft tissue defects.

Few studies have evaluated pulmonary function after chest wall reconstruction. Lardinois et al. [20] evaluated 26 patients with 3 to 8 rib defects, of which 39% underwent partial sternal resection at the same time. The sandwich technique with mesh, polymethylmethacrylate and mesh was used to reconstruct the chest wall. It was found that there was no significant difference in the forced expiratory volume at 1 s before the operation and 6 months after the operation. It was also found that 92% of patients exhibited consistent chest wall motion on magnetic resonance imaging.

The perioperative mortality of huge tumor resection in the chest wall is approximately 2—7% [21]. Respiratory and circulatory instability may occur during and after the operation, with lung-related complications and incision complications being the most common. We will continue to improve the reconstruction technology and perioperative management to receive the best effect.

## Conclusions

Huge tumors of the chest wall are a challenge for clinicians. Multidisciplinary discussion is generally needed before the operation. Surgeons should have a clear understanding of the anatomical structure around the tumor, formulate a careful and detailed resection plan, and achieve negative margins, which is very important for the long-term survival of patients. It is more reasonable and safer to choose a reconstruction method using mesh and titanium mesh. The latissimus dorsi flap could achieve good results in repairing soft tissue defects. It is also important to provide close perioperative management to ensure that these patients can achieve better curative effects.

## Data Availability

Not.
